# John Odlin Page

**Published:** 1888-08

**Authors:** 


### JOHN ODLIN PAGE, OBITUARY.

A few weeks ago the Chicago papers contained the intelligence of the self-destruction of Captain Page, a well-known citizen, at the ripe age of sixty-five, who was never known to do a dishonorable act and never lacked for friends.

John Odlin Page was born of distinguished ancestors, in Hallowell Maine, about the year 1822. He was prepared for college at the Hallowell Academy and afterwards graduated from Bowdoin College, Maine. As a student, he was what his fellow-students called, a " whole soul generous fellow," readier with his dog and gun, than his Greek and mathematics. It is said that, owing to some breach of discipline his diploma stuck a little on the way, concerning which Page was more indifferent that the faculty anticipated, and, when at length he received it duly decorated, he tied it with its own ribbon, around his retrivers neck saying that the dog was more deserving of it than he.

Mr. Page afterwards read law with Henry W. Paine, the eminent Hallowell lawyer who subsequently removed to Boston, residing for the time in his family.

Being called upon to superintend the extensive business affairs of his father, Mr. Page acquired an intimate knowledge of the structure and management of steam machinery and during our civil war, was induced to accept the captaincy of a Mississippi Mail Steamer largely engaged in carrying cotton.

Captain Page was a thoroughly honest man and refused to avail himself of any of the irregular methods then so greedily seized upon by the unscrupleous, of adding to his own fortune.

From the close of the war to the period of his death, ill luck seemed to follow him and nearly all of his ventures resulted in loss. No man had stronger or more willing friends, but his pride was so great that he withheld from them a knowledge of his necessities, and in many cases refused their offers of capital in enterprises which he feared would result disastrously. Captain Page was a man of eminent ability, as the following brief adieu to the world will illustrate, written by his hand even after the deadly portion, which by his own volition had begun to do its destroying work. It was the despairing valedictory of a great soul.

"Thou art too slow in thy coming, thou grim messenger; thou dost delight to thrust thy unwelcome presence among those who dread thee, and who still cling to life's empty bubble. But I, who have so long courted thy coming, who have prayed that toppling walls might crush me, that the lightenings bolt might scathe me, anything to terminate an intolerable existence so close-chased by adversity; I am forced to go out and hasten our meeting, and thus stain my hands with my own blood.

Too long has Adversity like an untiring and relentless wolf-hound followed my path. Opportunities for escape have been offered, but they led through dark avenues and through devious by-ways. I have always led the chase in the open field, that my foot-prints might show beneath the broad light of day, that every step was fairly taken. All avails naught. I feel the hot breath of my pursuer close upon me. And now to thy embrace, Oh Death! I fly for refuge.

Chicago, March 19, 1888.

